# Response of circulating heat shock protein 70 and anti-heat shock protein 70 antibodies to catheter ablation of atrial fibrillation

**DOI:** 10.1186/1479-5876-11-49

**Published:** 2013-02-23

**Authors:** Jelena Kornej, Claudia Reinhardt, Jedrzej Kosiuk, Arash Arya, Gerhard Hindricks, Volker Adams, Daniela Husser, Andreas Bollmann

**Affiliations:** 1 Department of Electrophysiology, Heart Center Leipzig, Strümpellstr. 39, 04289, Leipzig, Germany; 2 Department of Cardiology, Heart Center Leipzig, Strümpellstr. 39, 04289, Leipzig, Germany

**Keywords:** Atrial fibrillation, Heat shock proteins, Autoantibodies, Catheter ablation, AF recurrence

## Abstract

**Background:**

This pilot study investigated the association between heat shock protein 70 (HSP70) and anti-HSP70 antibodies as well as their changes and rhythm outcome after atrial fibrillation (AF) catheter ablation.

**Methods:**

We studied 67 patients with AF (59±11 years, 66% male, 66% lone AF) undergoing catheter ablation. Circulating HSP70 and anti-HSP70 antibody levels were quantified using commercially available assays before and 6 months after catheter ablation. Serial 7-day Holter ECGs were used to detect AF recurrences.

**Results:**

At baseline, HSP70 was detectable in 14 patients (21%), but there was no correlation between clinical or echocardiographic variables and the presence or the level of HSP70. In contrast, patients with paroxysmal AF (n=39) showed lower anti-HSP70 antibodies (median [IQR] of 43 [28 – 62] μg/ml) than patients with persistent AF (n=28; 53 [41 – 85] μg/ml, p=0.035). Using multivariable regression analysis, AF type was the only variable associated with anti-HSP70 antibodies (Beta=0.342, p=0.008). At 6 months, HSP70 was present in 27 patients (41%, p<0.001 vs. baseline). Similarly, there was an increase of anti-HSP70 antibodies (48 [36 – 72] vs. 57 [43 – 87] μg/ml, p<0.001). AF recurrence rates were higher in patients with HSP70 increase ≥0.025 ng/ml (32 vs. 11%, p=0.038) or anti-HSP70 increase ≥2.5 μg/ml (26 vs. 4%, p=0.033).

**Conclusions:**

HSP70 and anti-HSP70 antibodies may – at least in part – be associated in the progression of AF and AF recurrence after catheter ablation.

## Background

Heat shock proteins (HSPs) are characterized as molecular chaperones and have important functions in the preservation and protection of cells and organs from stress and injury. The HSPs are subdivided into multimember families based on the molecular weights of the proteins encoded such as the HSP90, HSP70, HSP60 and small HSP families [1]. They seem to play a dominant role in mediating cytoprotective effects and limit necrosis of smooth muscle cells exposed to oxidative stress [2]. HSPs are typically regarded as intracellular, but elevated levels of circulating HSPs have been found under several conditions including congestive heart failure, vascular disease or after acute myocardial infarction [3-6].

HSPs have also been implicated in the pathogenesis of atrial fibrillation (AF). In particular, myocardial HSP27 has been suggested to have protective effects against the progression from paroxysmal to persistent AF [7] or HSP70 against postoperative AF [8,9]. In contrast, circulating HSPs have different functions and may act as cytokines [10]. However, there is only few data available on circulating HSP levels in AF [8,11,12], although they may also be related with AF development [12]. Of interest, antibodies against various HSPs have also been associated with postoperative AF [13,14].

Catheter ablation has become the cornerstone of non-pharmacologic AF treatment, but AF recurrences are frequently observed and their pathophysiology is poorly understood. While pulmonary vein reconduction is the dominant mechanism [15], the possible contribution of ablation-induced tissue necrosis with subsequent development of inflammation and auto-immune reactions needs further investigation.

Consequently, this pilot study was performed to elaborate the potential role of soluble HSP70 (HSPA1A) [16] and anti-HSP70 antibodies in the AF development by (1) comparing plasma levels of patients with paroxysmal and persistent AF with AF-free controls and (2) by evaluating their response to catheter ablation that generates myocardial injury and their possible association with rhythm outcome.

## Methods

### Study population

This study enrolled 67 consecutive patients who underwent left atrial catheter ablation for drug-refractory paroxysmal or persistent AF (Table 1) and 34 controls matched for age, gender and heart disease. Patients with hematologic, renal, or hepatic impairment, systemic inflammation, neoplastic disorders, acute infection or thyrotoxicosis were excluded. Paroxysmal and persistent AF was defined according to current guidelines [17]. Paroxysmal AF was defined as self-terminating within 7 days after onset documented by previous ECG or Holter-ECG. Persistent AF was defined as an AF episode either lasting longer than 7 days or requiring drug or direct current cardioversion for termination.

**Table 1:** Baseline clinical, echocardiographic, and laboratory data of the study population

	**AF population**	**Controls**
	**n=67**	**n=34**
Age (years)	59 ± 11	59 ± 11
Males (%)	66	66
Paroxysmal AF (%)	58	0
Lone AF (%)	66	-
AF history (months)	72 ± 60	-
LVEF (%)	60 ± 9	62 ± 8
LAD (mm)	43 ± 6	41 ± 5
anti-HSP70 antibodies (μg/ml)	48 [36 – 72]	40 [32 – 70]
HSP70 (ng/ml)*	0 [0 – 0]	0 [0 – 0]
hs-CRP (μg/ml)	2.07 ± 1.10	1.80 ± 0.85

HSP70 and anti-HSP70 antibodies are presented as median [IQR].

* detectable in 21% of AF patients and 19% of controls.

LAD = left atrial diameter, LVEF = left ventricular ejection fraction.

Controls without AF-related symptoms were recruited from the outpatient clinic and freedom from AF was verified by previous ECG reports.

In all patients, transthoracic and transesophageal echocardiography was performed prior to catheter ablation. Left atrial diameter and left ventricular ejection fraction were determined using standard measurements and a left atrial thrombus was excluded. All class I or III antiarrhythmic medications with the exception of amiodarone were discontinued at least 5 half-lives before the procedure. The study was approved by the local ethics committee (Medical Faculty, University Leipzig) and patients provided written informed consent for participation.

### Catheter ablation

Left atrial catheter ablation was performed using a previously described approach [18]. In brief, patients were studied under deep propofol sedation with continuous invasive monitoring of arterial blood pressure and oxygen saturation. Non-fluoroscopic 3D catheter orientation, CT image integration, and tagging of the ablation sites were performed using Ensite NavX, Ensite Velocity (St. Jude Medical, St. Paul, MN, USA) or CARTO 3 (Biosense Webster, Diamond Bar, CA, USA). Trans-septal access and catheter navigation were performed with a steerable sheath (Agilis, St. Jude Medical., St. Paul, MN, USA). Patients presenting with AF at the beginning of the procedure were electrically cardioverted and ablation was performed during sinus rhythm (i.e. AF termination with ablation was not attempted). In all patients circumferential left atrial ablation lines were placed around the antrum of the ipsilateral pulmonary veins (irrigated tip catheter, pre-selected tip temperature of 48°C, and maximum power of 30 – 50 W). In patients with persistent AF, additional linear lesions were added at the left atrial roof, the basal posterior wall and the left atrial isthmus. Ablation of complex fractionated electrograms was not performed.

After circumferential line placement, voltage and pace mapping along the ablation line were used to identify and close gaps. The isolation of all pulmonary veins with bidirectional block was verified with a multipolar circular mapping catheter and was defined as the procedural endpoint.

### Follow-up

After ablation, class I and III antiarrhythmic drugs were not reinitiated. Oral anticoagulation was prescribed for 6 months, and proton pump inhibitors were added for 4 weeks. All patients were followed in the outpatient clinic for 6 months after the ablation. During this follow-up period, 7-day Holter recordings were performed 3 and 6 months after the ablation. Additional ECGs and Holter recordings were obtained when patients’ symptoms were suggestive of AF. AF recurrence was defined as a documented AF episode lasting longer than 30 seconds between 3 and 6 months after the ablation (thus, including a 3-month “blanking period”). All patients with sustained early recurring AF underwent direct cardioversion. Additional drug administration was left to the discretion of the treating physician.

### Blood samples

Blood samples were obtained before and 6 months after catheter ablation. Platelet-poor plasma fractions were obtained by centrifugation at 20°C for 10 min at 3500 × g, and plasma were stored at −80°C for subsequent analysis.

Plasma levels of HSP70, anti-HSP70 antibodies and high-sensitiveC-reactive protein (hs-CRP) were quantified using commercially available specific enzyme-linked immunoabsorbent assays (ELISA) according to the manufacturers protocol (Hsp70 High Sensitivity EIA Kit - Enzo Life Sciences ADI-EKS-715; anti-human Hsp70 (total) ELISA Kit - Enzo Life Sciences ADI-EKS-750; Human High Sensitivity C-Reactive Protein, hs-CRP ELISA Kit - Cusabio CSB-E08617h). Results were compared with standard curves and the lower detection limits were as follows: HSP70 0.09 ng/ml; anti-HSP70-autoantibodies 6.79 ng/ml; hs-CRP 0.08 μg/ml. The interassay and intra-assay precisions were < 10%.

### Statistical analysis

Statistical analysis was performed according to the following analysis plan. (1) HSP70 and anti-HSP70 antibodies were compared between AF patients and AF-free controls. (2) Possible associations between clinical or echocardiographic characteristics and HSP70 or anti-HSP70 antibodies were analyzed for the entire population and then for the AF cohort. (3) Changes in HSP70 and anti-HSP70 antibodies following catheter ablation were evaluated and (4) associations between those changes and clinical, echocardiographic or procedural characteristics were determined. (5) Patient characteristics including HSP70 and anti-HSP70 antibody levels and changes were compared between patients with and without recurring AF.

Continuous variables are reported as mean ± one standard deviation or median with interquartile ranges (IQR), and categorical variables are reported as frequencies. Continuous variables were compared using Mann–Whitney-U-test (unpaired data), Wilcoxon test (paired data) or ANOVA with post-hoc analysis and categorical variables were compared using the chi-square test. Multivariable analysis that included variables with a p-value <.1 found in univariate analysis was performed to identify independent predictors of baseline HSP70 and anti-HSP70 antibody levels as well as of AF recurrence.

Bonferroni correction for multiple comparisons was not used and a p value of < .05 was considered statistically significant.

## Results

### HSP70 and anti-HSP70 antibodies in AF

At baseline, HSP70 was detectable in 21% of the patients and in 19% of the controls (p=ns). Patients with AF and AF-free controls showed similar HSP70, anti-HSP70 antibody and hs-CRP levels (Table 1). There was no correlation between clinical or echocardiographic variables and the presence or the level of HSP70 in both the entire and the AF cohort. However, in the AF cohort patients with paroxysmal AF had lower anti-HSP70 antibodies (median 43, IQR 28 – 62 μg/ml) at baseline compared to patients with persistent AF (53, 41 – 85 μg/ml, p=0.035). Using uni- and multivariable regression analysis adjusted for age and gender, AF type was the only variable associated with anti-HSP70 antibodies (Beta=0.342, p=0.008). There was no correlation between HSP70, anti-HSP70 antibodies and hs-CRP at baseline.

### Response of HSP70 and anti-HSP70 antibodies to catheter ablation

Total ablation time was 40 ± 18 min and total energy was 77.279 ± 34.675 J. At 6 months after catheter ablation, HSP70 was present in 27 patients (41%, p<0.001 vs. baseline). While both the median and the lower quartile remained at 0 ng/ml the upper quartile increased from 0 to 0.09 ng/ml (p=0.029). Similarly, there was an increase of anti-HSP70 antibodies (48 [36 – 72] vs. 57 [43 – 87] μg/ml, p<0.001) while hs-CRP remained unchanged (2.07 ± 1.1 vs. 2.15 ± 1.2 μg/ml, p=0.425).

There was no relation between clinical, echocardiographic or procedural variables and anti-HSP70 and CRP levels after 6 months or their changes. In contrast, in patients with a HSP70 increase in the upper tertile (≥0.025 ng/ml), total ablation time was longer (50 ± 24 vs. 36 ± 14 min, p=0.012) and ablation energy was higher (98.889 ± 42.298 vs. 69.103 ± 27.813 J, p=0.024) compared to patients in the lower and intermediate tertile. Also, patients with newly detectable HSP70 at 6 months had a longer total ablation time (62 ± 23 vs. 35 ± 14 min, p<0.001) and a higher total ablation energy (117.056 ± 40.993 vs. 68.108 ± 27.193 J, p<0.001) compared to patients who had no HSP70 detectable at baseline and after 6 months.

There was no correlation between HSP70, anti-HSP70 antibodies and hs-CRP at 6 months.

### Response of HSP70 and anti-HSP70 antibodies and rhythm outcome after catheter ablation

AF recurrence between 3 and 6 months occurred in 18% of the patients. None of the baseline clinical, echocardiographic or laboratory variables was associated with AF recurrence, but ablation time and energy were higher in patients with recurring AF (Table 2). AF recurrence rates were higher in patients with HSP70 increase ≥0.025 ng/ml (32% vs. 11%, p=0.038) or anti-HSP70 increase ≥2.5 μg/ml (26 vs. 4 %, p=0.033). When both variables were entered in multivariable analysis, HSP70 changes in the upper tertile were the only independent predictor of AF recurrence resulting in an odds ratio of 3.733 (95% CI 1.026 – 13.589, p=0.046). However, when ablation time and energy were added to the model, total ablation time was the only independent predictor of AF recurrence (OR 1.088, 95% CI 1.034 - 1.144, p=0.001).

**Table 2:** Comparison of baseline clinical, echocardiographic, laboratory and procedural variables between patients with and without AF recurrence after 6 months

	**No AF recurrence**	**AF recurrence**
	**n=55**	**n=12**
Age (years)	59 ± 11	61 ± 12
Males (%)	63	75
Paroxysmal AF (%)	59	50
Lone AF (%)	69	58
AF history (months)	73 ± 62	68 ± 52
Body mass index (kg/m2)	28 ± 4	29 ± 5
Statins (%)	26	33
ACE inhibitor/Angiotensin receptor blocker (%)	69	50
LVEF (%)	60 ± 9	56 ± 11
LAD (mm)	43 ± 6	45 ± 9
Total ablation duration (min)	35+/−12 *	61+/−24 *
Total ablation power (J)	67.470 ± 23.732*	117.497 ± 44.058*
anti-HSP70-antibodies (μg/ml)	49, 35 – 73	47, 37 – 56
HSP70 (ng/ml)	0 [0 – 0]	0 [0 – 0.29]
hs-CRP (μg/ml)	2.08 ± 1.18	2.03 ± 0.61

* p<0.05.

Abbreviations and data presentation as in Table 1.

## Discussion

### Main findings

To the best of our knowledge, this study is the first to investigate the potential role of circulating HSP70 and anti-HSP70 antibodies in AF and their response to catheter ablation. Several findings are of special importance:

1) HSP70 and anti-HSP70 antibodies were similar between controls and AF patients, but anti-HSP70 antibody levels were associated with AF type, i.e. patients with persistent AF had higher anti-HSP70 antibody titers than their counterparts with paroxysmal AF;

2) HSP70 and anti-HSP70 antibodies increased after AF catheter ablation and the HSP70 increase was associated with total ablation time and energy, i.e. patients with newly detectable HSP70 or highest HSP70 increases had longer ablation times and higher ablation energies;

3) The increase of HSP70 and anti-HSP70 antibodies was associated with more frequent AF recurrences after 6 months in univariate but not in multivariable analysis that revealed total ablation time as sole predictor for AF recidivism.

### Heat shock proteins and antibodies in the progression from paroxysmal to persistent AF

The rate of progression from paroxysmal to persistent or permanent AF varies between 8% [19] and 22% [20] after 1 year but responsible mechanisms remain elusive. Heart failure, previous stroke or TIA, obstructive lung disease, hypertension, and age have been identified as independent clinical predictors of AF progression [21]. It has been postulated that underlying cardiovascular diseases such as heart failure and hypertension cause chronic atrial stretch and dilatation, which in turn seem to be important for atrial structural and electrophysiological remodeling supporting the initiation and maintenance of AF [22].

Myocardial HSPs, i.e. HSP27 or HSP70 may have several protective effects that prevent AF progression [7] or development of postoperative AF [8,9]. HSP expression may effect angiotensin-II-induced fibrosis [23] or atrial myolysis [7]. In addition, HSPs are crucial for the maturation of hERG encoding the alpha subunit of the cardiac potassium current *IKr*[24]. Finally, HSPs mediate protective responses to oxidative stress, which results from mitochondrial metabolism dysfunction and further impairs both mitochondrial and contractile function [25]. Taken together, HSPs are involved in a variety of aspects of the atrial remodeling process including structural and electrophysiological remodeling as well as oxidative stress.

In our study, there were no differences in circulating HSP70 and anti-HSP70 antibody levels between AF patients and AF-free controls. In addition, there was no difference in soluble HSP70 between paroxysmal and persistent AF, but anti-HSP70 antibodies could be identified as predictor for AF type. A previous study revealed no correlation between atrial and soluble HSP70 [8]. Consequently, circulating HSP70 levels do not reflect myocardial HSP70 expression or function and it is not surprising that there were no associations with AF presence or AF type. However, this study is the first to suggest that an anti-HSP70 antibody-mediated immune response is a possible contributor to the progression of the remodeling process. In individuals with high antibody levels, autoimmune reactions may occur when these antibodies encounter cells expressing HSPs on their surface [26]. In fact, antibodies against HSP60 [14] or HSP65 [13] have been implicated in the development of postoperative AF although their exact mode of action is not known. In addition, previous studies have found auto-antibodies against M2-muscarinic acetylcholine and beta-1 adrenergic receptors to promote AF development [27,28].

### Heat shock proteins and antibodies in recurring AF after catheter ablation

Isolation of the pulmonary veins with or without creation of additional linear lesions is the cornerstone of today’s AF catheter ablation procedures which has also been applied in our study. Radiofrequency ablation itself creates a localized myocardial necrosis as reflected by an increase in troponin, creatine kinase and activation of the inflammatory cascade [29]. While so far, no study has assessed the release of HSPs after radiofrequency catheter ablation, HSP70 release after myocardial infarction has been reported. Of interest, there was a correlation between the extent of myocardial infarction expressed by peak troponin or creatine kinase and HSP70, the latter being persistent for several weeks after the injury [3,5]. This observation is in support of our finding on the relation between HSP70 increase and ablation time and energy, even 6 months after catheter ablation.

Circulating HSPs act differently than myocardial HSPs and have a role in the systemic inflammatory response against myocardial injury induced by myocardial infarction or – as in our case – by catheter ablation. In fact, HSP70 functions not only as a molecular chaperone, but also as an activator of the immune system capable of inflammatory cytokine production [10]. HSP70 activates the Toll like receptor-4 (TLR4) signalling pathway and, as an endogenous natural adjuvant, dose-dependently induces the downstream production of inflammatory cytokines [30]. Inflammation in turn has been linked with recurring AF after cardioversion [31] and catheter ablation [32]. In other words, HSP70 release supports the inflammatory response after catheter ablation, although this response was not captured with hs-CRP in our study. In addition, both myocardial HSP70 release and expression may activate the formation of antibodies which is concordant with the observed increase in anti-HSP70 antibodies. Of interest, in univariate analysis the upper tertiles of both HSP70 and anti-HSP70 antibodies were associated with higher AF recurrence rates. However, in multivariable analysis, only total ablation time was predictive of AF recurrence indicating more complex ablation procedures as strongest determinant. This finding does however not rule out a role of auto-immunology processes in mediating AF initiation which has been suggested by previous investigations on postoperative AF [8,9].

### Study limitations

This study included a highly-selected patient population, i.e. patients referred for catheter ablation had drug-refractory AF in most cases. Consequently, it is not known whether the proposed AF progression via activation of anti-HSP70 antibodies is specific for refractory AF or can be found in the general AF population.

Although different HSPs and anti-HSP antibodies have been implicated in the AF pathophysiology, this study was limited to the analysis of circulating HSP70 and anti-HSP70. In addition, this study included only two measurement points, allowing no conclusion regarding the time course of marker changes. The source of circulating HSP70 is unknown but the association with ablation time and energy makes myocardial release and consecutive activation of auto-antibodies the likely mechanism although disease progression can not be ruled out. Finally, this analysis was restricted to the additional investigation of hs-CRP and did not include other markers of cardiac damage or inflammation.

The associations between anti-HSP70 antibodies and AF type as well as HSP70 and anti-HSP70 antibody increases and AF recurrence after ablation may point to parallel phenomena or imply causality which remains to be clarified although spontaneous variation can not be ruled out with certainty since repeat measurements in the control group were not available. In that respect, our study should be viewed as hypothesis generating and more mechanistic work is necessary to explore the evolving field of autoimmunology in AF. This area of investigation seems of particular clinical interest for AF prevention as HSP function may be induced and auto-immune responses suppressed [33].

Monitoring of AF recurrence was limited to serial 7-day Holter ECGs which is in line with current guidelines but this strategy may nevertheless have missed asymptomatic AF recurrences.

Finally, Bonferroni correction for multiple comparisons was not used with possible confounding effects.

In summary, this pilot study is highly exploratory, and would be better conceived as an initial report aiming to demonstrate feasibility and raise further hypotheses.

## Conclusions

HSP70 and anti-HSP70 antibodies may – at least in part – be associated in the progression of AF and AF recurrence after catheter ablation. Additional studies are warranted to further explore their role in these settings.

## Competing interests

The authors declare that they have no competing interests.

## Authors’ contributions

JK conceived of the study, and participated in its design and coordination and drafted the manuscript. CR carried out the immunoassays. JKos conceived of the study. AA and GH conceived of the study, helped to draft the manuscript. VA has made substantial contribution to interpretation of data. DH participated in its design and coordination and secured funding. AB conceived of the study, and participated in its design and coordination, performed the statistical analysis and helped to draft the manuscript. All authors read and approved the final manuscript.

## Funding sources

This study was supported in part by grants from the Volkswagen Foundation, Germany to Dr. Husser (grant numbers I/81545 and 84901). Publication fees were paid by the Heart Center Leipzig, Germany.
